# Liquid-Phase Exfoliation of Bismuth Telluride Iodide (BiTeI): Structural and Optical Properties of Single-/Few-Layer Flakes

**DOI:** 10.1021/acsami.2c07704

**Published:** 2022-07-25

**Authors:** Gabriele Bianca, Chiara Trovatello, Attilio Zilli, Marilena Isabella Zappia, Sebastiano Bellani, Nicola Curreli, Irene Conticello, Joka Buha, Marco Piccinni, Michele Ghini, Michele Celebrano, Marco Finazzi, Ilka Kriegel, Nikolas Antonatos, Zdeněk Sofer, Francesco Bonaccorso

**Affiliations:** † Graphene Labs, Istituto Italiano di Tecnologia, via Morego 30, 16163 Genova, Italy; ‡ Dipartimento di Chimica e Chimica Industriale, Università degli Studi di Genova, via Dodecaneso 31, 16146 Genoa, Italy; § Dipartimento di Fisica, Politecnico di Milano, Piazza Leonardo da Vinci 32, 20133 Milano, Italy; ∥ BeDimensional S.p.A., via Lungotorrente Secca 30R, 16163 Genova, Italy; ⊥ Department of Physics, University of Calabria, Via P. Bucci cubo 31/C Rende, Cosenza 87036, Italy; # Functional Nanosystems, Istituto Italiano di Tecnologia, via Morego, 30, 16163 Genova, Italy; ¶ Nanochemistry Department, Istituto Italiano di Tecnologia, via Morego 30, Genova 16163, Italy; ∇ Department of Inorganic Chemistry, University of Chemistry and Technology Prague, Technická 5, 16628 Prague 6, Czech Republic

**Keywords:** two-dimensional materials, liquid-phase exfoliation, Rashba effect, nonlinear optics, second-harmonic generation

## Abstract

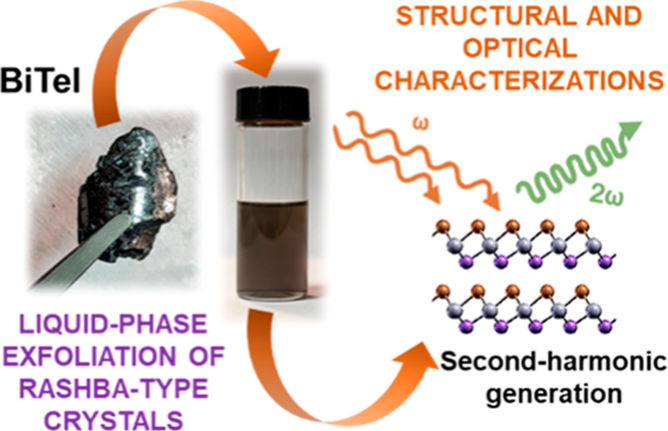

Bismuth telluride halides (BiTeX) are Rashba-type crystals with several potential applications ranging from spintronics and nonlinear optics to energy. Their layered structures and low cleavage energies allow their production in a two-dimensional form, opening the path to miniaturized device concepts. The possibility to exfoliate bulk BiTeX crystals in the liquid represents a useful tool to formulate a large variety of functional inks for large-scale and cost-effective device manufacturing. Nevertheless, the exfoliation of BiTeI by means of mechanical and electrochemical exfoliation proved to be challenging. In this work, we report the first ultrasonication-assisted liquid-phase exfoliation (LPE) of BiTeI crystals. By screening solvents with different surface tension and Hildebrandt parameters, we maximize the exfoliation efficiency by minimizing the Gibbs free energy of the mixture solvent/BiTeI crystal. The most effective solvents for the BiTeI exfoliation have a surface tension close to 28 mN m^–1^ and a Hildebrandt parameter between 19 and 25 MPa^0.5^. The morphological, structural, and chemical properties of the LPE-produced single-/few-layer BiTeI flakes (average thickness of ∼3 nm) are evaluated through microscopic and optical characterizations, confirming their crystallinity. Second-harmonic generation measurements confirm the non-centrosymmetric structure of both bulk and exfoliated materials, revealing a large nonlinear optical response of BiTeI flakes due to the presence of strong quantum confinement effects and the absence of typical phase-matching requirements encountered in bulk nonlinear crystals. We estimated a second-order nonlinearity at 0.8 eV of |χ^(2)^| ∼ 1 nm V^–1^, which is 10 times larger than in bulk BiTeI crystals and is of the same order of magnitude as in other semiconducting monolayers (e.g., MoS_2_).

## Introduction

Layered ternary bismuth telluride halides (BiTeX, X = Cl, Br, or I) have recently gained research interest because of their polar non-centrosymmetric or non-symmorphic structures coupled with sizeable spin–orbit interaction (SOI) effects,^1−5^ resulting from the mixed ionic–covalent character of the compound^6^ and the presence of heavy Bi atoms.^2^ Such distinctive features lift the Kramer’s spin degeneracy of both the three-dimensional (3D) bulk^1,2,7−9^ and two-dimensional (2D) surface states.^3,10−19^ This leads to the momentum-dependent spin splitting in the band structure in the absence of an external magnetic field, originating from both bulk and structural inversion asymmetries (i.e., Dresselhaus and Rashba effects), as described by a 2D electron gas Rashba–Bychkov model^20^ or its phenomenological extensions.^21,22^ Ab initio calculations of the band structure and angle-resolved photoemission spectroscopy studies on BiTeX have shown that direct consequences of the Rashba effect are complex Fermi surfaces^23−25^ and other related physical phenomena. The most intriguing ones are multiple-frequency Shubnikov–de Haas oscillations,^24,26−29^ temperature-robust Dirac Landau level structures,^30^ spin-polarized magneto photocurrents,^31^ and pressure-induced topological quantum phase transitions toward non-trivial topological insulators with material side-dependent Dirac states.^28,29,32−37^ The evolution toward new quantum phases can lead to the fascinating pressure-dependent bulk photovoltaic effect^38^ and pressure-induced superconductivity.^33,34^ The surface state splitting off from the bulk bands in the presence of a potential change within the near-surface layer^3,10−14^ and the rational control of the spin information in BiTeX through the chemical potential-/doping-/mechanical stress-modulated Rashba effect represent new paradigm shifts in the realization of spin(orbi)tronic (spin devices,^1,10,39−44^ including spin field-/Hall-effect transistors,^22^ spin–orbit torque devices,^45^ spin injectors,^46^ and “Rashba p–n junctions”)^10,47^ as well as thermoelectric^48−51^ and piezoelectric systems^52^ with tunable nonlinear optical properties^53^ and even electrocatalytic materials.^54^

In this context, pioneering^1,10,23^^,^ and recent^22,55,56^ theoretical studies coupled with experimental observations^1^ revealed that BiTeI exhibits a giant Rashba spin splitting of hundreds of meV (i.e., ∼400 meV),^1^ which is among the largest reported so far. Its layered structure is built of ionically bound (BiTe)^+^ and I^–^ layers,^6,52^ forming BiTeI trilayers that are held together by van der Waals forces.^6,52^ Consequently, the plane between Te and I is a natural cleavage plane of the crystal, revealing Te- or I-terminated surfaces.^10,16,17^ Along with the rapid advances in designing artificial van der Waals heterostructures by stacking 2D materials,^56−58^^,^ exfoliated BiTeI flakes might be architectural components in functional quantum systems,^59−61^ including the realization of time-reversal invariant topological insulating phases,^62,63^ such as the predicted Bi_2_Te_2_I_2_ sextuple layer composed by Te-faced BiTeI.^64^ The realization of 2D materials with giant Rashba effects is a fascinating strategy to enable nanometer-scale spintronics operating at room temperature,^65−67^ as well as nanoscale piezoelectric^68^ and nonlinear optical applications.^69^ More generally, thanks to their morphology and the plethora of their distinctive properties, 2D materials are foreseen as the ultimate building blocks to produce high-energy density (opto-) electronic devices with radically new functionality, aiming at expressing the “More than Moore” vision.^70−72^

Despite the layered structure of the bulk BiTeI and the predicted low cleavage energy to obtain its monolayer (*ca.* 90 meV/atom), which is comparable to the one reported for transition metal chalcogenides^73−75^ and only *ca.* 60 meV/atom larger than the one of graphite,^76^ the exfoliation of BiTeI in the single-/few-layer form remains challenging.^77^ For example, BiTeI monolayers have been obtained through stripped gold exfoliation.^78^ However, this methodology leads to a strong hybridization of the BiTeI monolayer with the Au substrate,^78^ causing substantial modifications of the surface charge distribution onto the BiTeI surface.^78^ Recently, multi-layer BiTeI flakes with thickness down to 10 nm and marginal topological etching of the iodine atoms have been produced through electrochemical exfoliation in *N*,*N*-dimethylformamide (DMF) with the aid of tetrabutylammonium or lithium cations.^54^ For further advancement of the production and processing of the material class of 2D BiTeX, we report here for the first time the ultrasonication-aided liquid-phase exfoliation (LPE) of contamination-free BiTeI crystals. Notably, the exfoliation of Rashba-type layered polar crystals in liquid media expands the portfolio of solution-processed 2D materials, providing a novel type of functional ink for large-scale, high-speed, and cost-effective device manufacturing.^79−84^

In this work, the LPE of BiTeI crystals and the material dispersion stabilization are studied in 12 different solvents, allowing the Hildebrand and Hansen parameters of the BiTeI to be estimated. In fact, the material exfoliation yield is maximized by minimizing the Gibbs free energy of the solvent/crystal mixture.^85−87^ Experimentally, this condition corresponds to matching the solubility parameters of the solvent with those of the crystal.^85−87^ Moreover, it is important to identify a portfolio of different solvents for the exfoliation and processing of layered crystals.^79−81^ In particular, the properties of the solvents (e.g., viscosity, vapor pressure, and boiling points, just to name a few) can impact the characteristics of the films obtained by casting/printing the exfoliated crystal dispersion to such an extent as to impede/allow eligible deposition techniques.^87,88^ Our results indicate that the solvents with a surface tension (γ^sol^) close to 28 mN m^–1^ (surface energy of 68 mJ m^–2^) and a Hildebrandt parameter (δ_Hild_) between 19 and 25 MPa^0.5^ minimize the Gibbs free energy of the mixture solvent/BiTeI crystal.^85,89^ In addition, the use of low-boiling point and high-vapor pressure solvents effectively avoids solvent residuals while eliminating the substrate interaction occurring during stripped gold exfoliation.^78^ The morphological, structural, optical, and chemical properties of the exfoliated BiTeI flakes are evaluated here through a combination of microscopic and spectroscopic techniques, including nonlinear optical microscopy for second-harmonic generation (SHG). In fact, the concurrent spin–orbit coupling and structural inversion asymmetry of Rashba-type materials manifest themselves in nonlinear optical signals whose characteristics can be defined or even correlated to the Rashba strength of the material.^90,91^ Consequently, 2D Rashba-type materials potentially provide novel nanometer-thin platforms for nonlinear optical studies and applications.^91−93^ In addition, as shown for other polar 2D materials, such as group-IV metal monochalcogenides,^94^ the lack of inversion symmetry and strong quantum confinement can lead to extraordinary second-order nonlinear optical effects.^94^ Even more, since the nanometric thickness of exfoliated materials is much smaller than the second-harmonic (SH) coherence length, 2D materials bypass phase-matching constraints encountered in 3D nonlinear crystals.^95,96^ Thus, we measure SHG from few-layer BiTeI flakes, which exhibit a large nonlinear optical response 10-fold more intense than that of bulk BiTeI crystals and of the same order of magnitude as that of group VI monolayer transition metal dichalcogenides (TMDs) (|χ^(2)^| ∼0.1–1 nm V^–1^).^95,97^ These results prove the potential of LPE-produced BiTeI as a solution-processable low-dimensional Rashba-type material.

## Methods

### Crystal Synthesis and Exfoliation

The crystals of BiTeI are synthesized by direct reaction of the atomic elements (Bridgman method), according to previous protocols.^54^ Experimentally, stoichiometric amounts of bismuth, tellurium, and iodine (total weight of 6 g) are placed in a quartz glass ampule and sealed under high vacuum with an oxygen–hydrogen torch. Liquid nitrogen cooling is used during sealing to avoid iodine loss. The ampule is heated at 650 °C with a 1 °C min^–1^ heating rate, and after 6 h, it is cooled down to 400 °C with a 0.2 °C min^–1^ cooling rate. Finally, it is treated for 7 days at 400 °C and cooled down to room temperature overnight.

### LPE of BiTeI Crystals

The BiTeI flakes are produced by LPE of bulk crystals,^75,98−102^ followed by sedimentation-based separation^74,103−106^ to remove unexfoliated material. In more detail, 50 mg of powdered bulk crystals are added to 50 mL of anhydrous solvents and ultrasonicated in a bath sonicator (Branson 5800 cleaner, Branson Ultrasonics) for 15 h. Various solvents are investigated, including water, isopropanol (IPA), acetonitrile, ethanol, methanol, *N*-methyl-2-pyrrolidone, *n*-hexane, DMF, chloroform, chlorobenzene, ethylene glycol, and acetone. The as-produced dispersions are ultracentrifuged at 700*g* (Optima XE 90 with a SW32Ti rotor, Beckman Coulter) for 20 min at 15 °C. Then, 80% of the supernatant is collected by pipetting, thereby obtaining the dispersions of the exfoliated materials.

### Material Characterization

Scanning electron microscopy (SEM) imaging of the BiTeI crystals is carried out using a Optima XE 90 with a SW32Ti rotor), and elemental composition and mapping of the materials are obtained using an energy-dispersive X-ray spectroscopy analyzer (X-Max^N^) with a 20 mm^2^ Si drift detector (X-Max^N^ SDD, Oxford Instruments) and the AZtecEnergy software. The measurements are performed using an electron beam in the range 5–10 kV. The samples are prepared by placing powdered crystals directly on a carbon conductive tape.

Bright-field transmission electron microscopy images of BiTeI flakes are acquired with a JEM 1011 (JEOL) transmission electron microscope TEM), equipped with a thermionic W filament operating at 100 kV. The samples are produced by depositing the BiTeI flake dispersion in IPA onto ultrathin C-on-holey C-coated Cu grids. The samples are rinsed with deionized water and subsequently dried overnight under vacuum before measurements. High-resolution TEM (HRTEM) characterization is performed using a JEOL JEM2200 image-corrected microscope operated at 200 kV and equipped with an in-column Omega energy filter and a Bruker Quantax 400 EDX system with a 60 mm^2^ XFlash detector. The samples for these observations are prepared by drop-casting the dispersion of BiTeI flakes in IPA onto ultrathin C-coated Cu grids.

The atomic force microscopy (AFM) images are acquired with a NX10 AFM (Park System, Korea) by means of a non-contact cantilever PPP-NCHR 10 M (Nanosensors, Switzerland) having a tip diameter inferior to 10 nm, a resonance frequency of ∼330 kHz, and a force constant of 42 N m^–1^. The images are collected in the non-contact mode on an area of 5 × 5 μm^2^ (1024 × 1024 data points), keeping the working setpoint above 70% of the free oscillation amplitude. The scan rate for the acquisition of the images is 0.2 Hz. The samples are prepared by drop-casting a 1:10 diluted dispersion of BiTeI flakes in IPA onto mica sheets (G250-1, Agar Scientific Ltd.) and heating to 100 °C for 15 min to dry the sample and remove adsorbates.

Absorbance spectroscopy measurements are performed on the dispersions of exfoliated BiTeI flakes with a Cary Varian 5000 spectrophotometer using quartz glass cuvettes. The as-produced dispersions of the exfoliated materials are diluted with the corresponding solvents at different ratios to determine the extinction coefficient of the BiTeI flakes. The extinction coefficient is determined by using the Beer–Lambert law, that is, *A* = α_ext_*cL*, where *A* is the absorbance at 700 nm, α_ext_ is the extinction coefficient, *c* is the concentration of the exfoliated materials, and *L* is the optical path length (1 cm).

Diffusive reflectance spectroscopy (DRS) measurements are performed using a Cary Varian 5000 spectrophotometer with an integrating sphere on films of BiTeI flakes deposited by spray coating onto quartz substrates. The diffusive reflectance (*R*) data are analyzed according to the Kubelka–Munk theory.^107^ Experimentally, the optical band gap (*E*_g_) is estimated by fitting the linear part of [*F*(*R*)*h*ν]^*b*^ versus *h*ν (Tauc Plot) with [*F*(*R*)*h*ν]^*b*^ = *K*(*h*ν – *E*_g_) (Tauc relation), where *F*(*R*) is the Kubelka–Munk function, defined as *F*(*R*) = (1 – *R*)^2^/2*R*, where *h* is Planck’s constant, ν is the photon’s frequency, and *K* is a proportionality constant.^107^ The value of *b* indicates the type of electronic transitions differentiating between direct (*b* = 2) and indirect interband transitions (*b* = 0.5).^108^ According to the electronic structure of the BiTeI bulk^1,10^ and monolayer,^60,61,68^*b* = 0.5 is considered for the analysis of our sample.

X-ray diffraction (XRD) measurements are acquired with a PANalytical Empyrean using Cu Kα radiation. The samples for XRD are prepared by depositing powder of the BiTeI bulk crystal or BiTeI flakes (from IPA dispersion) onto Si/SiO_2_ substrates.

Raman spectroscopy measurements are performed using a Renishaw microRaman inVia 1000 mounting an objective with 0.9 numerical aperture (NA), using an excitation wavelength of 514 nm and an incident power of 1 mW. For each sample, 50 spectra are collected to assess the reproducibility of the data. The samples are prepared by drop-casting the as-prepared BiTeI flakes dispersion onto Si/SiO_2_ substrates and subsequently dried under vacuum.

Nonlinear optical measurements are performed on exfoliated BiTeI flakes drop-casted onto a SiO_2_/Si substrate. The optical excitation is provided with a soliton mode-locked Er:Yb:glass laser (Onefive, Origami 15–80) emitting pulses of 160 fs duration and a 1551 nm center wavelength with a repetition rate of 80 MHz. The pump beam is focused onto the sample through a 0.85 NA dry objective (Nikon, CFI Plan Fluor 60XC) to a diffraction-limited illumination spot of about 1.8 μm diameter. The nonlinear emission is collected through the same objective in a back-scattering configuration, spectrally filtered at the SH wavelength (Figure S4), and detected using a single-photon avalanche diode (Micro Photon Devices, PD-050-CTD).

### Statistical Analysis

Gwyddion 2.60 software was used to process the height profiles of the flakes imaged by AFM, while ImageJ software (NIH) was used to analyze the lateral size of the flakes imaged by BF-TEM. The lateral size of a flake was estimated as the mean of maximum and minimum lateral sizes of the flakes. OriginPro 9.1 software was also used to carry out the statistical analysis of the thickness and lateral size data. 300 BiTeI flakes on multiple AFM and TEM images were considered for the statistical analysis of the thickness and lateral size, respectively, of the BiTeI flakes.

## Results and Discussion

**Figure 1:**
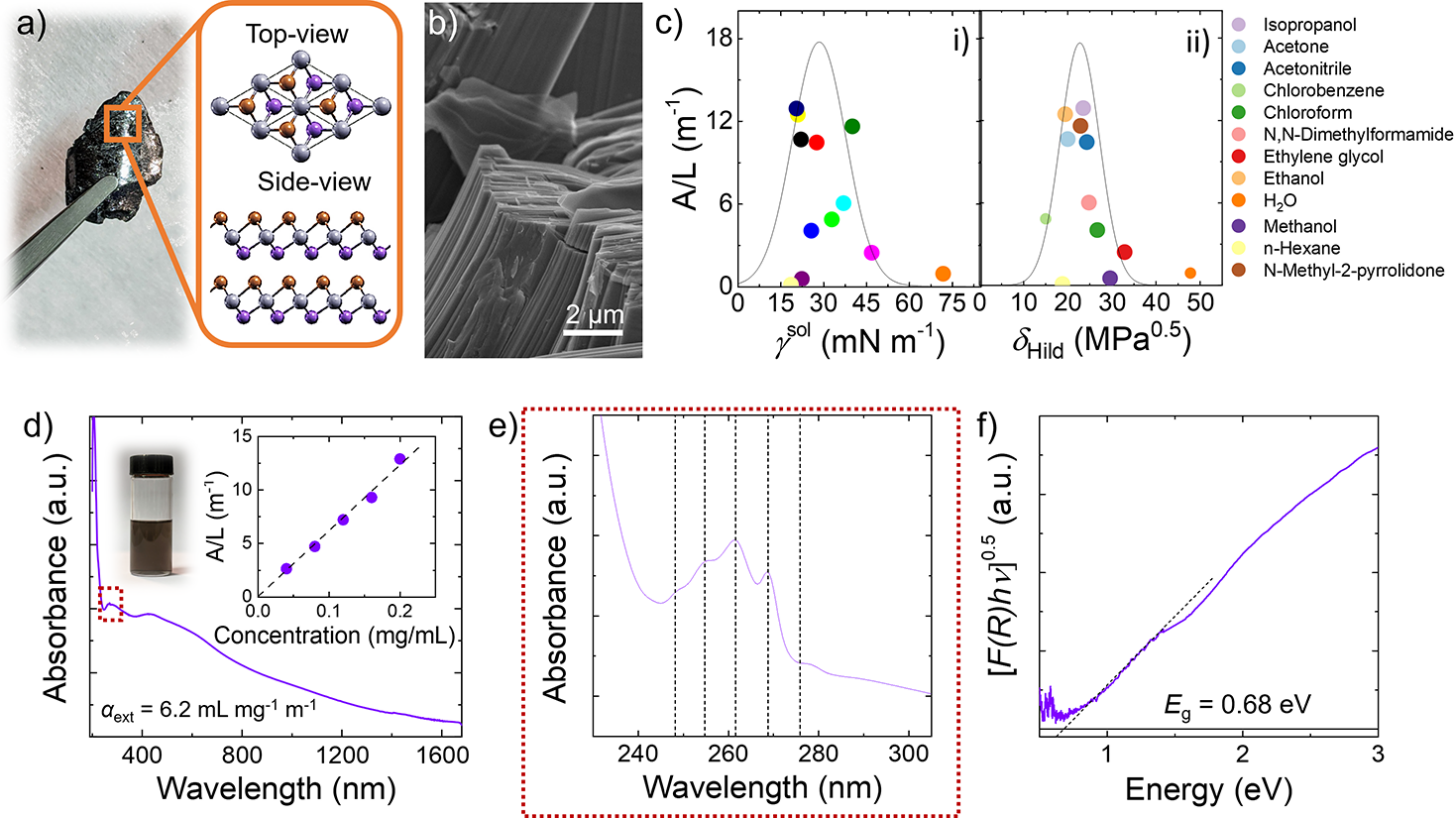
(a) Photograph of a BiTeI crystal produced through direct synthesis of its elements. The crystal structure (space group *P*3*m*1, no. 156) of the BiTeI crystals is also shown. (b) SEM image of a fragment of the BiTeI crystal, evidencing its layered structure. (c) Concentration (plotted as *A*/*L*) of the BiTeI flake dispersion produced through LPE dispersed in different solvents, plotted versus solvent γ^sol^ (panel i) and δ_Hild_ (panel ii). The continuous lines are Gaussian fits to data. (d) Absorbance spectra of the BiTeI flake dispersion in IPA. The photograph of the dispersion is also shown. The inset shows the Lambert–Beer plot of the BiTeI dispersion. (e) Absorbance spectra of the BiTeI flake dispersion in IPA in the ultraviolet, evidencing the fine structure of the optical transitions at high energy. (f) Tauc plot of a film of BiTeI flakes produced by spray coating the BiTeI flake dispersion in IPA onto a quartz substrate.

Hexagonal (space group *P*3*m*1, no. 156) BiTeI crystals are produced through direct synthesis of their elements, following protocols described in the literature (see the Methods section).^54^Figure 1a shows a photograph of a representative BiTeI crystal, together with its layered polar crystal structure built of ionically bound (BiTe)^+^ and I^–^ layers (i.e., trilayers with I–Bi–Te stacking) tied together by van der Waals forces.^6,78^Figure 1b shows the SEM image of fragments of BiTeI crystals, whose layered structure is visible at their edges. The as-produced BiTeI crystals are further characterized by SEM-coupled energy-dispersive X-ray spectroscopy (EDS) (see Supporting Information, Figure S1), showing a nearly ideal BiTeI stoichiometry of 0.96:0.98:1. According to the predicted cleavage energy to obtain its monolayer (*ca.* 90 meV/atom) by cleaving the crystals along the Te–I planes, we initially investigate the LPE of the BiTeI bulk crystals by the prototypical ultrasonication method.^79,103,109^ To further elucidate the exfoliation of BiTeX materials, we perform the ultrasonication-assisted LPE of the as-synthesized BiTeI crystals in 12 different solvents with diverse values of surface tension (γ^sol^) and/or solubility parameters. Notably, the Gibbs free energy of the mixture solvent/layered material must be minimized to maximize the exfoliation of layered crystals and stabilize the resulting dispersion of exfoliated materials.^85−87,89^ This condition is accomplished when the surface free energy of the material is equal to the surface free energy of the solvent, which can be calculated from γ^sol^, that is,^89^ γ^sol^ = *E*_surf_^sol^ – TS_surf_^sol^, where *E*_surf_^sol^ is the solvent surface energy, *T* is the absolute temperature, and S_surf_^sol^ is the solvent surface entropy (∼10^–3^ J m^–2^ K^–1^).^89^ Experimentally, the matching of the Hildebrand or Hansen solubility parameters of the layered materials with the ones of the solvent promotes the exfoliation processes.^85,86,110^ The Hildebrand parameter (δ_Hild_) is defined as the square root of the cohesive energy density, that is, δ_Hild_ = [(Δ*H*_v_ – *RT*)/*V*_m_]^0.5^, where Δ*H*_v_ is the enthalpy of vaporization, *R* is the ideal gas constant, and *V*_m_ is the molar volume.^86^ Based on empirical considerations, solvents with δ_Hild_ values within a “small” range (e.g., within ±2 MPa^0.5^ in polymer science) centered at the δ of the material are considered good solvents.^111^ However, the δ_Hild_-matching condition does not account for hydrogen bonding and polar interactions,^86^ which can drastically affect the material solubility/dispersibility in a solvent.^112^ Contrary to the Hildebrand model, the Hansen model predicts the solubility/dispersibility of material by referring to three parameters, which are the dispersion, polar, and hydrogen bonding components (δ_D_, δ_P_, and δ_H_, respectively) of δ_Hild_, as expressed by δ_Hild_^2^ = δ_D_^2^ + δ_P_^2^ + δ_H_^2^.^86,111^ The Hansen parameters can be visualized in a 3D plot with 2δ_D_, δ_P_, and δ_H_ axes, resulting in a point for each solvent or material. According to the Hansen solubility criterion, suitable solvents are those which fall within a “small” sphere (e.g., a sphere with radius ≤8 MPa^0.5^ in polymer science) centered at a point corresponding to the material.^111^ Therefore, by plotting the extinction coefficients of the LPE-produced BiTeI dispersion as a function of γ^sol^ or the solubility parameters of the corresponding solvents, it is possible to estimate the surface free energy of the layered crystals, as well as their solubility parameters, from the maximum of the data distribution.^85−87^Figure 1c shows the *A*/*L* versus γ^sol^ and *A*/*L* versus δ_Hild_ plots, respectively, where *A* is the absorbance and *L* is the cell length. Being *A*/*L* = α_ext_*c* (α_ext_ is the extinction coefficient, and *c* is the concentration of the exfoliated material, see the Methods section), the BiTeI exfoliation is maximized for solvents with γ^sol^ close to 28 mN m^–1^ (surface energy of 60 mJ m^–2^) and δ_Hild_ between 19 and 25 MPa^0.5^. This means that the surface free energy and δ_Hild_ of the BiTeI crystals are estimated within the range of values for the solvent capable of efficiently exfoliating the material. The plots of *A*/*L* versus Hansen parameters are shown in Figure S2. These data provide δ_P_, δ_H_, and δ_D_ values for BiTeI in the range of 6–17, 6–19, and 15–18 MPa^0.5^, respectively. The exfoliation in IPA yielded a maximum concentration of ∼0.2 mg mL^–1^. This means that the exfoliation yield, defined as the ratio between the weight of dispersed exfoliated flakes (after the centrifugation process, see details in the Methods section) and that of the starting bulk crystals (1 mg mL^–1^ concentration), is ∼20%. Figure 1d shows the absorption spectrum and the photograph of the BiTeI flake dispersion in IPA, which is stable over hundreds of hours (more than 1 month). The *A*/*L* plot (inset to Figure 1d) scales linearly with the concentration, allowing the estimation of α_ext_ at *ca.* 6.2 mL mg^–1^ m^–1^. The absorption spectrum shows a long tail toward the near-infrared (NIR) region, in agreement with previous literature on both bulk^8,113^ and exfoliated BiTeI. Absorption bands are observed around 435 nm (2.85 eV) and 265 nm (4.68 eV). These optical features have been almost disregarded in previous literature, which typically focused on the low-energy region (<1 eV).^8^ Notably, in an isolated Bi^3+^ ion, the 6s^2^ configuration exhibits the ^1^S_0_ ground state, while the 6s^1^ 6p^1^ configuration generates four excited states: three ^3^P_0,1,2_ states and one ^1^P_1_ state.^114^ The absorption band at 265 nm cannot be assigned either to the ^1^S_0_ → ^3^P_0,1,2_ spin-forbidden transitions or to ^1^S_0_ → ^1^P_1_ since 265 nm is not compatible with any wavelength at which those transitions are expected.^114−116^ Moreover, Figure 1e shows that this high-energy band is the overlap of at least five different contributions equally spaced by 0.13 eV. We speculate that such a band originates from the excitation of one electron from Bi^3+^ 5d orbitals to Bi^3+^ 6p orbitals. This excitation should produce several excited states in the Bi^3+^ ion, all having the same 5d^9^6s^2^6p^1^ electronic configuration. The fine structure of BiTeI flakes may depend on the crystalline field and SOIs and may also be influenced by the Rashba magnetic field. The *E*_g_ value of the BiTeI flakes is estimated through DRS using the Kubelka–Munk theory of diffusive reflectance (*R*) (see additional details in the Methods section).^107^Figure 1f shows the corresponding Tauc plot, considering BiTeI flakes as indirect band gap materials (i.e., in the Tauc relation described in the Methods section, *b* = 0.5). From the Tauc relation, the *E*_g_ of BiTeI flakes is estimated as 0.68 eV. This value is similar to those computed by DFT simulations for monolayer BiTeI (electronic transition near the Γ point),^60,61,68,78^ suggesting that our films are mainly made of single and few-layer flakes (see additional discussion hereafter). The low-energy (<*E*_g_) signal is attributed to intraband transitions occurring between spin-conduction bands, which involve electronic states with opposite spin orientations.^8,113^

**Figure 2:**
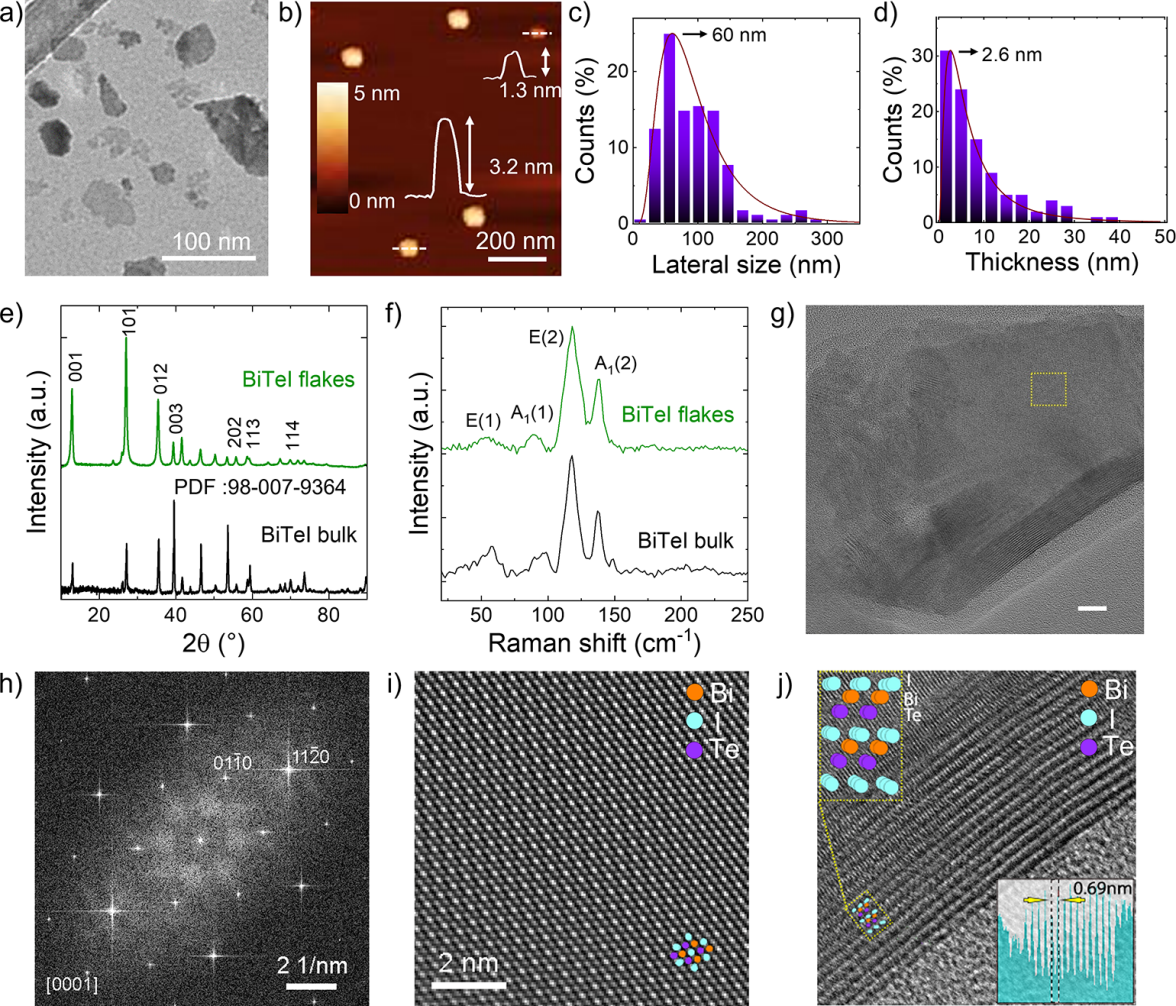
(a) BF-TEM and (b) AFM images of the BiTeI flakes produced through LPE of the BiTei crystal in IPA. (c) TEM statistical analysis of the lateral size of the BiTeI flakes (300 flakes). (d) AFM statistical analysis of the thickness of the BiTeI flakes (300 flakes). (e) XRD diffractograms and (f) Raman spectra (excitation wavelength of 514 nm) of BiTeI bulk crystals and flakes. The panels, respectively, report the diffraction peaks and the Raman modes attributed to the hexagonal *P*3*m*1 structure of the BiTeI crystals. (g) HRTEM image of a typical BiTeI flake with one edge folded onto itself (bottom bottom corner) and (h) the corresponding FFT confirming the single-crystalline nature of the flake and an exact [0001] orientation. (i) Close-up view of the area outlined by a rectangle in panel (g), showing the atomic arrangement as viewed from the [0001] direction. The atomic arrangement matches the expected atomic arrangement of BiTeI in the same orientation (a model is overlaid on top). (j) A folded edge of the flake reveals that it is 14 or 15 BiTeI unit cells thick. Individual monolayers spaced by 0.69 nm are clearly resolved.

The morphology of the LPE-produced BiTeI flakes in IPA is evaluated through AFM and TEM measurements. Figure 2a shows a TEM image of representative flakes with irregular shapes. Figure 2b reports an AFM image of representative flakes, whose height profiles correspond to flake thicknesses between 1 and 4 nm. The statistical analysis of the lateral size and thickness of the BiTeI flakes (Figure 2c,d) shows that the data follow a log-normal distribution peaked at ∼60 and ∼2.6 nm, respectively. These data indicate that the exfoliated sample mainly consists of single-/few-layer BiTeI flakes. Notably, previous studies reported an experimental AFM thickness of the BiTeI monolayer on the Au substrate in the range of 8.5 ± 1.2 Å.^78^ Since BiTeI monolayers strongly interact with Au (binding energy of 681 and 969 meV for I and Te-terminations of BiTeI, respectively),^78^ this value is close to bulk lattice parameters of BiTeI in the out-of-plane direction, that is, 6.5 Å or 6.9 Å.^6^ In our case, by considering the distance between BiTeI flakes and the mica substrate, the lowest measured thicknesses (∼1 nm) are attributed to BiTeI monolayers. The structural properties of the BiTeI flakes are evaluated by XRD and Raman spectroscopy. The XRD pattern of BiTeI flakes (Figure 2e) shows the same diffraction peak as that of the native bulk crystal, which matches the hexagonal *P*3*m*1 structure (PDF card: 98-007-9364).^6,49,54^ This means that the LPE process preserves the crystallinity of the starting crystals without bringing about any additional phase. Figure 2f shows the Raman spectra of both BiTeI flakes and bulk crystals. The group theory predicts four active Raman modes, with the irreducible vibrational representation Γ = 2Α_1_ + 2E. (i.e., two E modes and two A_1_ modes).^117,118^ The peaks at 90 and 138 cm^–1^ are assigned to A_1_(1) and A_1_(2) modes, respectively, while E(1) and E(2) are found at 58 and 118 cm^–1^. Contrary to previous studies on 2D BiTeI,^78,54^ the presence of distinguishable Raman peaks in our exfoliated sample indicates a high crystallinity of the BiTeI flakes, in agreement with the XRD analysis. The crystalline nature of the BiTeI flakes is further confirmed by HRTEM measurements. Figure 2g reports a HRTEM image of two flakes overlapped, with the one on the right folded onto itself. The corresponding fast Fourier transform (FFT) of the HRTEM image (Figure 2h) confirms the single-crystalline nature of the flake and an exact [0001] orientation, which indicates that the bulk BiTeI is exfoliated perpendicular to the *c* axis of its hexagonal crystal structure, as expected by its layered structure.^6^Figure 2i shows a close-up view of the area outlined by a rectangle in Figure 2g, revealing an atomic arrangement as viewed from the [0001] direction.

Such an atomic arrangement matches the one expected for BiTeI in the same orientation (see the model at the top left corner of panel j). The HRTEM image of the fold on the edge of the flakes (Figure 2j) reveals that the flake is 14 or 15 BiTeI unit cells thick, with the individual monolayers spaced at ∼0.69 nm clearly resolved. Figure S3 shows the high-angle annular dark-field–scanning transmission electron microscopy (HAADF–STEM) image of a BiTeI flake, together with the corresponding STEM–EDS maps of Bi, Te, and I. The quantitative elemental analysis results in a Bi:Te:I atomic ratio of 1:0.93:0.98 and a low atomic content of O (O:Bi atomic ratio of 0.17:1), which means that the LPE process in anhydrous IPA preserves the chemical integrity of the native crystal. It should also be noted that the use of IPA as a low-boiling point and high-vapor pressure solvent for the LPE process avoids solvent contamination of the flakes once deposited, as previously shown for other types of 2D materials (e.g., few-layer black phosphorous).^87^

**Figure 3:**
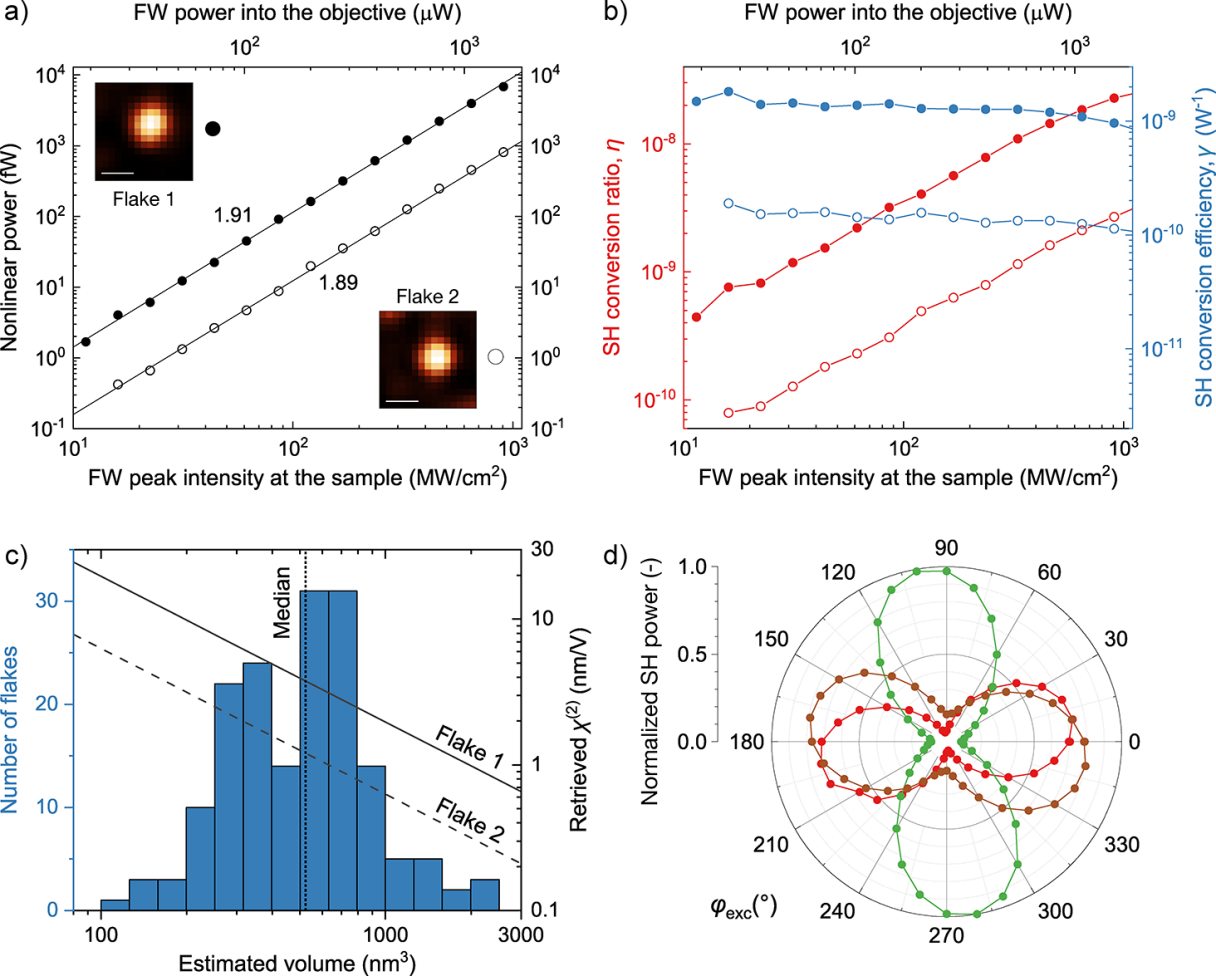
(a) SH power emitted by two few-layer BiTeI flakes (flake 1: solid dots and flake 2: empty dots) as a function of the FW peak intensity. Insets: confocal SH scans of the two flakes (the scale bar is 1 μm). The power law fits (solid lines, *P* ∝ *I*^*p*^) have an exponent *p* of 1.91 and 1.89, respectively. (b) Measured SH conversion ratio η (red) and conversion efficiency γ (blue) of the same two flakes as a function of the FW peak intensity. (c) (Histogram, left axis) retrieved number distribution of the flake volume; the median value of 524 nm^3^ is indicated by the vertical dotted line. The overlaid lines (solid and dashed correspond to flakes with full and hollow symbols of panel a, respectively) show the analytical model of  expressed by eq 1. (d) Normalized SH power emitted by representative (individual) few-layer BiTeI flakes as a function of the pump polarization direction. Different colors indicate different flakes.

To further confirm the non-centrosymmetric structure and crystalline quality of both bulk BiTeI and LPE-produced BiTeI crystals, their nonlinear optical properties are evaluated by SHG (Figures 3, S5, and S6). Figure 3 illustrates the SH emission by liquid phase-exfoliated BiTeI flakes drop-casted onto a SiO_2_/Si substrate under pulsed excitation at the 1550 nm telecom wavelength (C band). Note, however, that we are not targeting any specific geometric (e.g., Mie-type) or material (e.g., excitonic) resonance. Indeed, the absence of sharp spectral features around the pump and SHG wavelength in the absorption spectrum (Figure 1d), together with the lack of the phase-matching requirement at subwavelength thicknesses, suggests that the nonlinear conversion efficiency reported below does not disperse strongly and therefore is amenable to broadband operation.

Figure 3a shows the nonlinear emission measured at 775 nm on two few-layer BiTeI flakes (named flake 1 and flake 2) and the power law fits. The quadratic dependence of the nonlinear power on the excitation power is the fingerprint of the second-order nonlinear optical process. The reported SH power represents the power emitted by the sample and entering the microscope objective, which has been quantified by considering the optical transmission or efficiency of all the elements in the detection path. The observed sample photodamage threshold is ∼1 GW cm^–2^ (Figure S5). The third-harmonic generation (THG) signal is detectable only above the photodamage threshold (Figure S5), so its fluence dependence is not reliable. Figure 3b shows the SH conversion ratio (η) and the conversion efficiency (γ) as a function of the fundamental wavelength (FW) peak intensity. The conversion ratio is defined as η = *P*_SH_/*P*_FW_, where *P*_SH_ and *P*_FW_ are the instantaneous (pulse peak) powers of SH emission and FW, respectively. Notably, η is expected to depend linearly on *P*_FW_. The conversion efficiency is defined as γ = *P*_SH_/*P*_FW_^2^ and characterizes the nonlinear emission of the material itself, being independent of the excitation (neither its power nor the repetition rate). Compared to the bulk nonlinear emission, BiTeI flakes show a 10-fold stronger nonlinear response (Figure S6). Recently, bulk BiTeI has been shown to possess a second-order nonlinear susceptibility χ^(2)^ as large as ∼400 pm V^–1^. Therefore, by comparing the SH emission of our liquid-phase exfoliated BiTeI flakes with a native bulk, a |χ^(2)^| of the order of 1 nm V^–1^ is reasonably expected. To estimate the χ^(2)^ of the material in its exfoliated form, we modeled the few-layer flake as an electric dipole lying flat on the planar SiO_2_ surface. As described in Supporting Information, the analytical model accounts for the effect of the dielectric interface on the dipole radiation as well as for the finite solid angle of collection defined by the objective, leading to
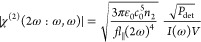
1Here, ε_0_ is the permittivity of vacuum, *c*_0_ is the speed of light in vacuum, *n*_2_ is the refractive index of air (equal to 1), *l*_∥_ is a coefficient depending on the refractive index ratio of the interface and assumes that the dipole is parallel to the surface itself, *V* is the volume of the flake, and *f* is the fraction of emitted power collected by the objective (defined as *P*_det_ = *fP*_tot_, where *P*_tot_ is the total SH power radiated by the dipole).

Now, the volume *V* of individual flakes is not known since AFM and TEM, as well as nonlinear optical measurements, are not correlated to single flakes. Nevertheless, to estimate the order of magnitude of , *V* can be retrieved by multiplying the TEM lateral size distribution (Figure 2c) with the median of the AFM thickness data (Figure 2d). Thus, Figure 3c reports  against the retrieved number distribution of *V*. According to eq 1, χ^(2)^ is inversely proportional to the *V* of the measured flake. By conservatively considering a large *V* of 1000 nm^3^, one obtains χ^(2)^ = 2 nm V^–1^ for flake 1 and 0.6 nm V^–1^ for flake 2. Such values are comparable to or larger than the typical susceptibilities of standard 3D semiconductors (e.g., 0.2 nm V^–1^ for GaAs) and similar to those previously reported for other 2D semiconductors in their monolayer form (e.g., 0.1–1 nm V^–1^ for TMDs).^95,97^ Note that the buried interface with the Si wafer below the 285 nm thick SiO_2_ substrate is not taken into account. Multiple reflections and interferences (i.e., Fabry–Pérot etaloning) contribute to the uncertainty affecting our estimate of |χ^(2)^|, which is, however, likely dominated by the uncertainty of *V*. Nevertheless, our results probe the potential of BiTeI, a representative layered Rashba-type material, as a nonlinear optical platform, both in its bulk and exfoliated form (i.e., single-/few-layer flakes).

As known from literature,^16^ BiTeI crystals typically suffer from stacking faults. The surfaces in correspondence with such stacking faults may represent natural cleavage planes that are preferably subjected to the exfoliation process, leading to flakes with mixed surface terminations. It is therefore reasonable that the quality of the starting BiTeI crystal could affect the final morphological properties (e.g., thickness and lateral size) and structural characteristics of the LPE-produced BiTeI flakes. In this context, the dependence of the emitted nonlinear optical power on pump linear polarization can be used as a tool to probe such structural defects.^119,120^ Although the nonlinear optical measurements are limited by diffraction to areas of ∼2 μm^2^, Figure 3d reveals that the SH power reaches its maximum value for well-defined impinging pump polarizations. A marked dependence of the SH emission on the linear pump polarization indicates a lack of mirror symmetry in the 2D lattice structure material, which can be found in the crystal structure of monolayer BiTeI or few-layer BiTeI flakes with the absence of stacking faults. Therefore, these data support that the monocrystalline phase of few-layer flakes is being measured. Conversely, Figure S7 indicates that some other emitters show two preferential excitation directions for SHG. These emitters might be BiTeI flakes with a stacking fault, as intrinsically occurring in bulk BiTeI crystals,^10,16,121^ as well as two distinct flakes within the laser spot. On the contrary, within the focused sample spot, the polarization response of multiple or aggregated flakes would be averaged to nearly isotropic due to their random orientations. Prospectively, the analysis of SHG dependence on the pump linear polarization may provide a valuable tool to evaluate the quality of both bulk and exfoliated BiTeI crystals, as well as other layered BiTeX.

## Conclusions

In summary, we report the first ultrasonication-assisted LPE of BiTeI crystals, serving as a model for layered Rashba-type materials. By screening 12 different solvents, we elucidate the importance of minimizing the Gibbs free energy of the solvent/BiTeI crystal mixture to maximize the BiTeI exfoliation and dispersion. In more detail, this condition is achieved for solvents with surface tension γ^sol^ close to 28 mN m^–1^ (surface energy of 60 mJ m^–2^) and the Hildebrand parameter (δ_Hild_) between 19 and 25 MPa^0.5^. By applying the Hansen solubility criterion, we estimate polar (δ_P_), hydrogen bonding δ_H_, and dispersion (δ_D_) parameters for BiTeI in the range of 6–17, 6–19, and 15–18 MPa^0.5^, respectively. Once the exfoliation of BiTeI crystals in the form of single-/few-layer flakes is optimized, we assess their morphological, structural, optical, and chemical characteristics through combined microscopic and spectroscopic techniques.

The non-centrosymmetric structure and crystallinity of both bulk and exfoliated BiTeI are further confirmed by their nonlinear optical response. Nonlinear frequency up-conversion of NIR light, demonstrated in this work, has high technological relevance as it enables detection with silicon-based devices. Together with the high-throughput solution-processed fabrication approach that we prove, our results suggest that exfoliated BiTeI can find application in functional inks and thin-film coatings for anti-counterfeiting schemes or night-vision devices. By modeling the BiTeI flakes as electric dipoles and determining their amplitude based on quantitative measurements, |χ^(2)^| is estimated. Our results show that few-layer BiTeI can exhibit a large second-order nonlinear optical response (χ^(2)^ ∼1 nm V^–1^), which is 10-fold more intense than that in bulk BiTeI crystals and possesses the same order of magnitude as that in other 2D materials in the monolayer form. Overall, our study provides guidelines for the LPE of Rashba-type polar BiTeX, highlighting its potential for advanced solution-processed applications, including spin(orbi)tronic, thermoelectric, piezoelectric, and nonlinear optical systems, as well as other energy conversion devices.

## Supporting Information Available

Supplementary SEM–EDS characterizations; Hansen parameter analysis; supplementary HAADF–STEM characterizations; additional details of the SHG and THG experimental setup; and supplementary SHG and THG characterizations (PDF)

## Abbreviations

2D: two-dimensional
3D: three-dimensional
AC: acetone
ACN: acetonitrile
AFM: atomic force microscopy
BF-TEM: bright-field transmission electron microscopy
CB: chlorobenzene
CF: chloroform
DMF: *N*,*N*-dimethylformamide
DRS: diffusive reflectance spectroscopy
EtOH: ethanol
EDS: energy-dispersive X-ray spectroscopy
EG: ethylene glycol
FFT: fast Fourier transform
FW: fundamental wavelength
HRTEM: high-resolution transmission electron microscopy
IPA: isopropanol
LPE: liquid-phase exfoliation
MeOH: methanol
NA: numerical aperture
NMP: *N*-methyl-2-pyrrolidone
SBS: sedimentation-based separation
SEM: scanning electron microscopy
SH: second harmonic
SHG: second-harmonic generation
TEM: transmission electron microscopy
THG: third-harmonic generation
TMDs: transition metal dichalcogenides
XRD: X-ray diffraction
