# Supplementary material for: Liquid-Phase Exfoliation of Bismuth Telluride Iodide (BiTeI): Structural and Optical Properties of Single-/Few-Layer Flakes
Source: ACS Appl Mater Interfaces. 2022 Jul 25;14(30):34963–74. doi: 10.1021/acsami.2c07704 (PMC9354013; doi:10.1021/acsami.2c07704)
Supplement: Supplementary file 1 — am2c07704_si_001.pdf [file am2c07704_si_001.pdf]

Supporting Information

Liquid-phase exfoliation of bismuth telluride iodide (BiTeI): structural and optical properties of single-/few-layer flakes

Gabriele Bianca,[1,2] Chiara Trovatello,[3] Attilio Zilli,[3] Marilena Isabella Zappia,[4,5] Sebastiano Bellani,[4]* Nicola Curreli,[6] Irene Conticello,[4] Joka Buha,[7] Marco Piccinni,[1,2] Michele Ghini,[2,6] Michele Celebrano,[3] Marco Finazzi,[3] Ilka Kriegel,[6] Nikolas Antonatos,[8] Zdeněk Sofer[8] and Francesco Bonaccorso[1,4]*

1 Graphene Labs, Istituto Italiano di Tecnologia, via Morego 30, 16163, Genova, Italy

2 Dipartimento di Chimica e Chimica Industriale, Università degli Studi di Genova, via Dodecaneso 31, 16146 Genoa, Italy

3 Dipartimento di Fisica, Politecnico di Milano, Piazza Leonardo da Vinci 32, 20133, Milano, Italy

4 BeDimensional S.p.A., via Lungotorrente Secca 30R, 16163, Genova, Italy

5 Department of Physics, University of Calabria, Via P. Bucci cubo 31/C Rende, Cosenza, 87036 Italy

6 Functional Nanosystems, Istituto Italiano di Tecnologia, via Morego, 30, 16163, Genova, Italy

7 Nanochemistry Department, Istituto Italiano di Tecnologia, via Morego 30, Genova, 16163 Italy

8 Department of Inorganic Chemistry, University of Chemistry and Technology Prague, Technická 5, Prague 6, 16628 Czech Republic

Corresponding Author

*s.bellani@bedimensional.it, *f.bonaccorso@bedimensional.it

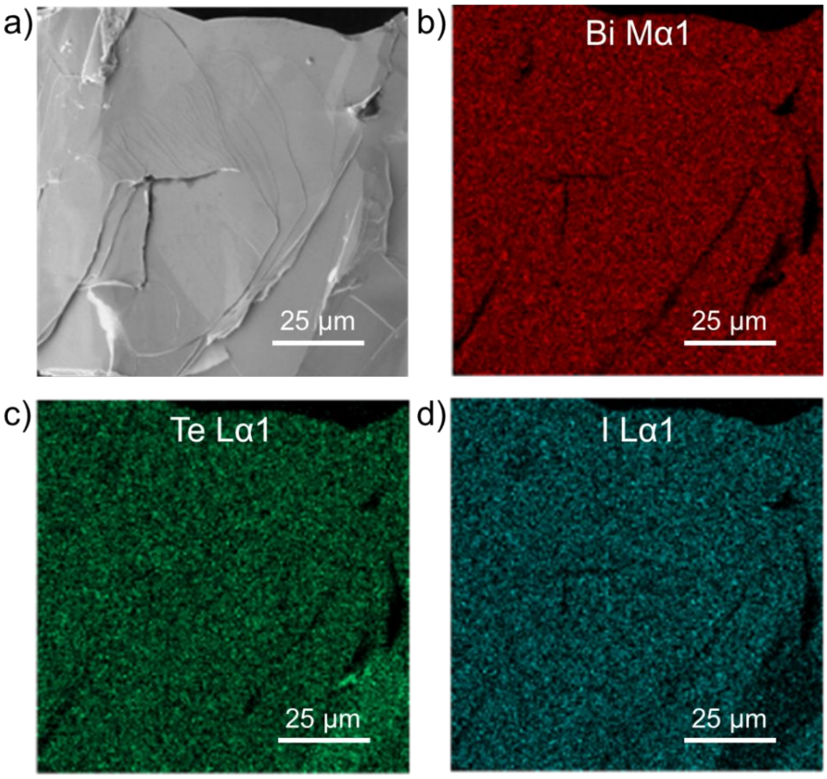

Figure S1. a) SEM image of a region of BiTeI crystals and b–d) the corresponding EDS maps for Bi (Mα1 line), Te (Mα1 line) and I (Lα1 line), respectively.

Figure S1 reports the scanning electron microscopy (SEM)-coupled energy-dispersive X-ray spectroscopy (EDS), which revealed a nearly ideal Bi:Te:I stoichiometry of 0.96:0.98:1.

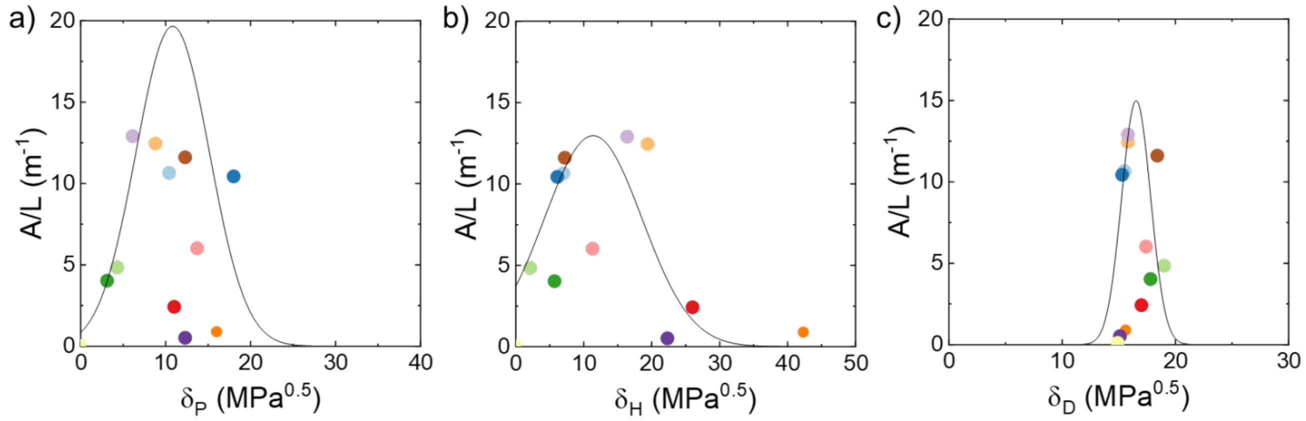

Figure S2. a-c) Concentration (plotted as A/L) of the BiTeI flakes dispersion produced through LPE in different solvents, plotted *vs.* solvent Hansen parameters (δ_D, $\delta_{P,}$ and δ_H).

Figure S2 reports the plots of A/L (in which A is the absorbance and L is the cell length) *vs.* solvent Hansen parameters, measured for the BiTeI flakes dispersion produced through LPE in different solvents. As discussed in the main text, these data provide an estimation of polar (δ_P), hydrogen bonding δ_H and dispersion (δ_D) values for BiTeI. The continuous lines are Gaussian fits to data.

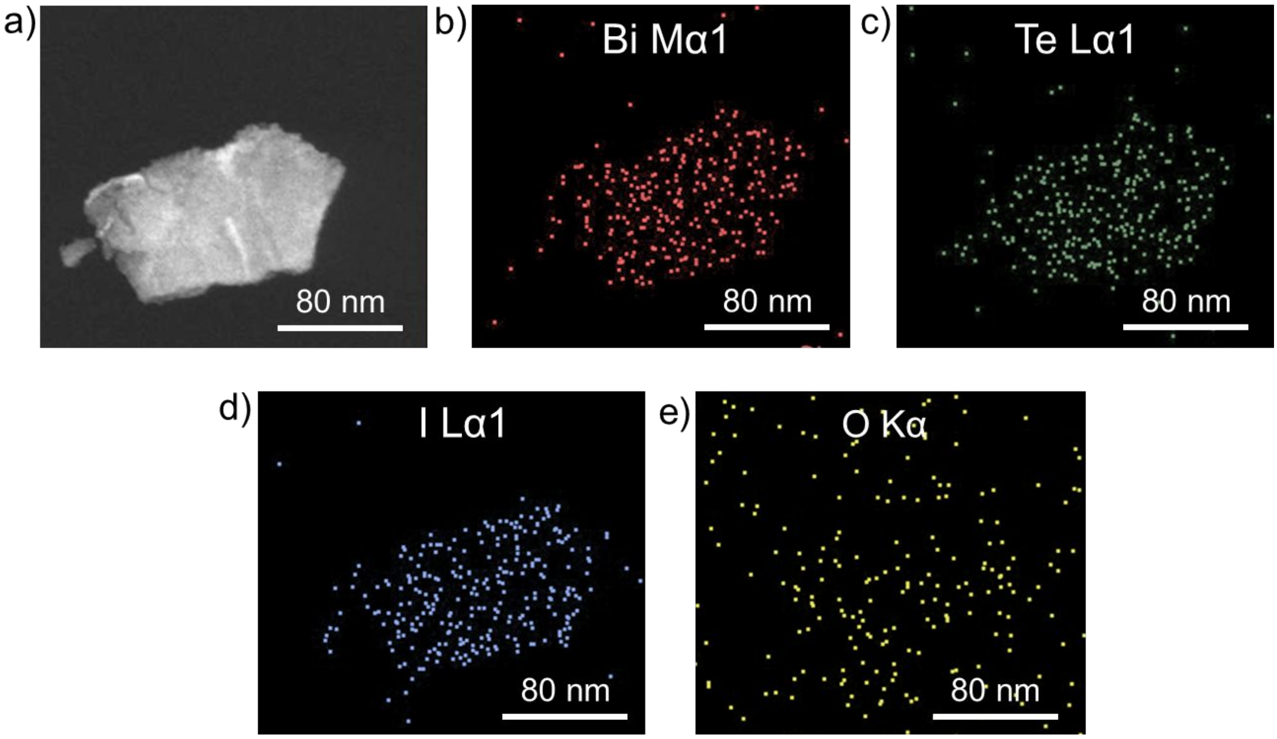

Figure S3. a) STEM image of a BiTeI flake and b-e) the corresponding quantitative STEM-EDS maps of Bi (Mα1 line), Te (Mα1 line), I (Lα1 line) and O (Kα line), respectively.

Figure S3 shows the high-angle annular dark-field STEM (HAADF-STEM) image of a BiTeI flake, together with the corresponding STEM-EDS maps of Bi, Te and I. The quantitative elemental analysis indicated a Bi:Te:I atomic ratio of 1:0.93:0.98 and a low atomic content of O (O:Bi atomic ratio of 0.17:1).

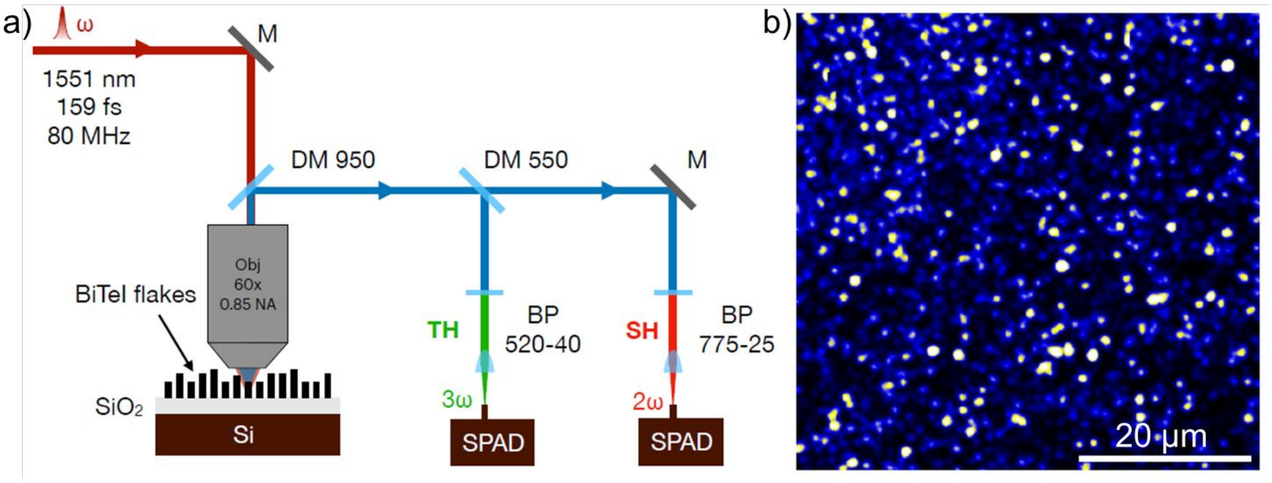

Figure S4. Second harmonic (SHG) and third harmonic generation (THG) experimental setup. a) Diagram of the nonlinear optical microscopy setup. M = metallic mirror, DM = dichroic mirror (cut-off wavelength indicated, in nm), BP = bandpass filter (centre wavelength-bandwidth indicated, in nm), SPAD = single-photon avalanche diode. b) Normalized SHG power map measured on LPE-produced BiTeI flakes deposited on a SiO_2/Si substrate. The scale bar is 20 µm.

The nonlinear microscope used for second harmonic generation (SHG) and third harmonic generation (THG) characterization is shown in **Figure S4a**. The source of the nonlinear optical measurements is an ultrafast laser emitting 160 fs pulses at 1551 nm with a repetition rate of 80 MHz. The linearly polarized excitation beam is coupled to a dry 60x objective with NA=0.85 (Nikon, CFI Plan Fluor 60XC) resulting in a diffraction-limited focus diameter on the sample of about 1.8 µm. The nonlinear emission is collected by the same objective in a backscattering geometry and the reflection and fluorescence of the pump is filtered by a long pass (cut-off wavelength 950 nm) dichroic mirror (DM). The nonlinear signal is then separated by a second long pass (cut-off wavelength 550 nm) DM in two beams. Each beam is spectrally filtered by two sharp edge bandpass filters at 775 nm (bandwidth 40 nm) and 520 nm (bandwidth 25 nm), *i.e.*, the SH and the TH wavelengths, enabling their simultaneous detection onto two single-photon avalanche diodes (SPADs), whose small sensor area effectively implements a confocal detection scheme. The sample can be raster scanned under the pump beam by an xyz sample stage (P517.3CL, Physik Instrumente) to produce spatial maps such as that in **Figure S4b**. The average lateral size of the flakes is below 100 nm, therefore the sample inhomogeneities (darker regions) can be attributed to solvent aggregates that can form if the solvent evaporates rapidly during the sample preparation.

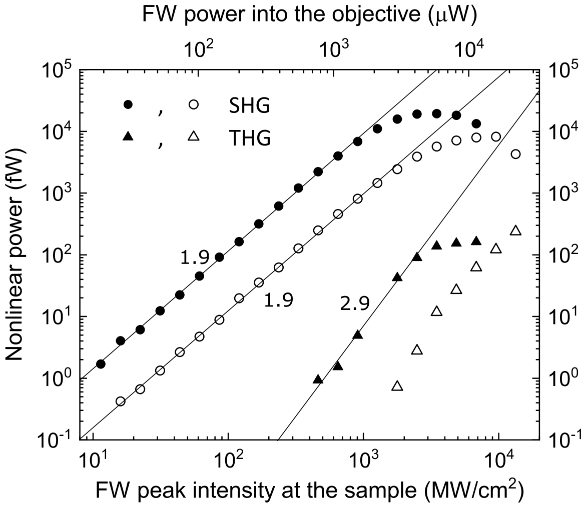

Figure S5. SHG and THG emission from few-layer BiTeI flakes. SH (circle) and TH (triangle) power as a function of the fundamental wavelength (FW) peak intensity emitted by two randomly selected few-layer BiTeI flakes (full and hollow symbols, respectively). The power law fits (solid lines, $P \propto I^p$) of SH and TH signals have an exponent p of 1.9 and 2.9, respectively.

Figure S5 shows the dependence on the pump power on the nonlinear emission already reported in **Figure 3** of the main text, here in the full measured range and including THG data. The dependencies are fitted by power laws (solid lines) whose exponent is reported near each line. The experimental data of SH (circle) and TH power (triangle) follow a quadratic and cubic dependence on the pump power, respectively, which are fingerprints of second- and third-order nonlinear optical processes. The linear trend starts deviating at ~ 1 GW cm^{-2}, which is the estimated photodamage threshold. Note that for the second flake (hollow symbols) THG is detectable only above the photodamage threshold, and therefore its power dependence is not reliable.

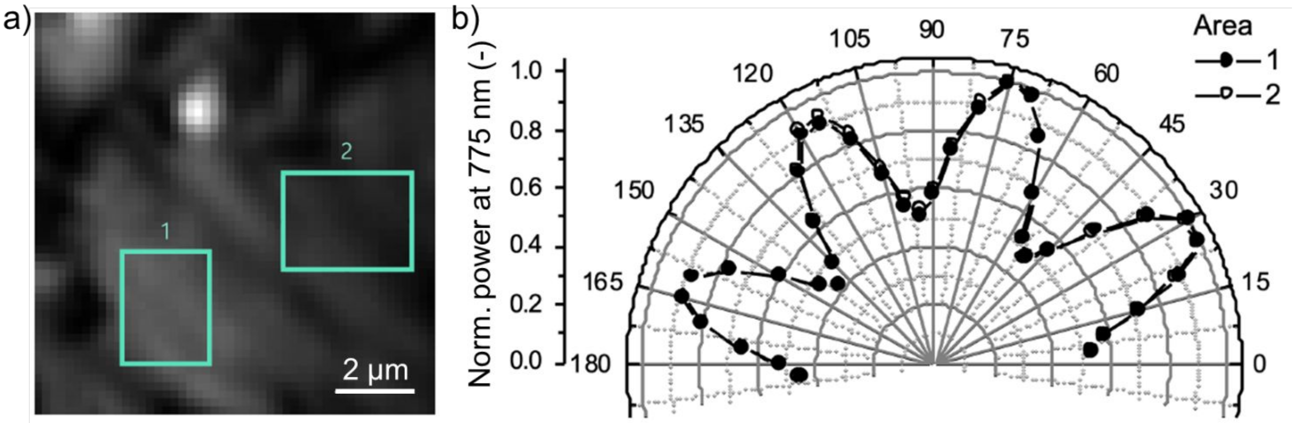

Figure S6. SHG from bulk BiTeI. a) Normalized SHG intensity map of bulk BiTeI excited with a pump intensity ~0.6 GW cm^{-2}. b) Normalized SHG intensity as a function of the pump polarization direction measured on two different areas of bulk BiTeI (turquoise frames in panel a).

In order to quantify the SHG intensity from LPE-produced BiTeI flakes, control measurements are performed on a fragment of a bulk BiTeI crystal. A flat and relatively clean-looking area on the bulk sample is chosen for the nonlinear imaging (**Figure S6a**). **Figure S6b** shows the normalized SH emission from two areas as a function of the pump polarization angle. Each data point is taken as the mean pixel value of the two selected areas marked in **Figure S6a**. Different areas show the same SH emission dependence on the pump polarization given by the symmetry of the $\chi^{(2)}$ tensor and the orientation of the crystal structure with respect to the optical axis of the microscope. Maximum SHG intensity for area 1 (2) is 1.9x10^5 (9.2x10^4) counts/s. In comparison, under the same excitation intensity of 0.6 GW cm^{-2}, a representative few-layer BiTeI flake (named flake 1 in the main text) emits 1.2x10^6 counts/s, about 1 order of magnitude more than the bulk sample.

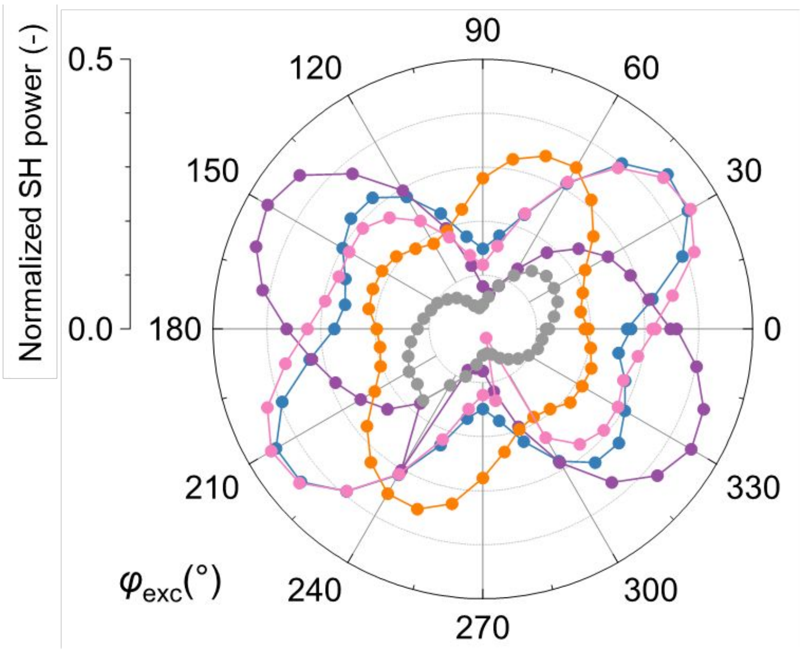

Figure S7. Normalized SH power emitted by few-layer BiTeI flakes as a function of the pump polarization direction, showing a polarization diagram with four lobes. Different colors indicate different flakes.

Figure S7 reports the normalized SH power emitted by few-layer BiTeI flakes as a function of the pump polarization direction, corresponding to a polarization diagram with four lobes. Such emitting flakes might be either BiTeI flakes with a stacking fault, as commonly observed in native bulk BiTeI crystals, or two distinct flakes within the laser spot.

To provide an estimate of the $\chi^{(2)}$ of the material in its exfoliated form, we modeled the flake as an electric dipole lying flat on the planar SiO_2 surface. As described in Supporting Information, the analytical model accounts for the effect of the dielectric interface on the dipole radiation as well as for the finite solid angle of collection defined by the objective. The electric dipole $\vec{p}(2\omega)$ oscillates at the SH frequency 2ω and has a amplitude

$$p(2\omega) = \varepsilon_0 |\chi^{(2)}(2\omega{:}\omega,\omega)| \, |\vec{E}(\omega)|^2 V \#(S1)$$

where ε_0 is the permittivity of vacuum, $\vec{E}(\omega)$ is the pump electric field, and V is the volume of the flake. The latter can be directly estimated from the AFM/TEM microscopic characterizations of the flake population, and $\vec{E}(\omega)$ results from the known pump intensity on the sample ($I(\omega)$), being $I(\omega) = \frac{1}{2}\varepsilon_0 c_0 n_2 |\vec{E}(\omega)|^2$ (approximately, the excitation on the flake is a plane wavefront since the lateral size of the flake is much smaller than the laser spot). The only unknown left to determine $|\chi^{(2)}|$ from Eq. (S1) is therefore $p(2\omega)$, which can be retrieved from the detected power. On the air side, $\vec{p}(2\omega)$ lies flat on the SiO_2–air interface. The radiation pattern of an electric dipole placed on a dielectric interface, as described in refs. [1,2,3], can be integrated over the solid angle defined by the acceptance of the objective to estimate the fraction f of the total nonlinear radiation that is actually collected. Being the refractive indices of the media for the SiO_2 substrate (n_1) and for the air (n_2) equal to 1.47 and 1.00, respectively, and the NA of the objective equal to 0.85, f is calculated to be 0.13, since most of the emission is towards the denser medium (namely, the SiO_2 substrate). The total SH power radiated by the dipole is

$$P_{tot}(2\omega) = \frac{n_2 \, l_{\parallel} \, (2\omega)^4}{12\pi\varepsilon_0 c_0^3} p(2\omega) \#(S2)$$

where c_0 is the speed of light in vacuum, $l_{\parallel}$ is a coefficient depending on the refractive index ratio of the interface and assumes that the dipole is parallel to the surface itself. Since the collected power is defined as $P_{det} = fP_{tot}$, one has the following equation

$$|\chi^{(2)}(2\omega{:}\omega,\omega)| = \sqrt{\frac{3\pi\varepsilon_0 c_0^5 n_2}{f \, l_{\parallel} \, (2\omega)^4}} \frac{\sqrt{P_{det}}}{I(\omega) \, V} . \#(S3)$$

References

(1) Lukosz, W.; Kunz, R. E. Fluorescence Lifetime of Magnetic and Electric Dipoles near a Dielectric Interface. *Opt. Commun.* **1977**, *20* (2), 195–199.

(2) Lukosz, W.; Kunz, R. E. Light Emission by Magnetic and Electric Dipoles Close to a Plane Interface. I. Total Radiated Power. *J. Opt. Soc. Am.* **1977**, *67* (12), 1607–1615.

(3) Lukosz, W.; Kunz, R. E. Light Emission by Magnetic and Electric Dipoles Close to a Plane Dielectric Interface. II. Radiation Patterns of Perpendicular Oriented Dipoles. *J. Opt. Soc. Am.* **1977**, *67* (12), 1615–1619.
